# The Uptake, Transfer, and Detoxification of Cadmium in Plants and Its Exogenous Effects

**DOI:** 10.3390/cells13110907

**Published:** 2024-05-24

**Authors:** Xintong Zhang, Man Yang, Hui Yang, Ruiqi Pian, Jinxiang Wang, Ai-Min Wu

**Affiliations:** 1 State Key Laboratory for Conservation and Utilization of Subtropical Agro-Bioresources, Guangdong Key Laboratory for Innovative Development and Utilization of Forest Plant Germplasm, College of Forestry and Landscape Architecture, South China Agricultural University, Guangzhou 510642, Chinarqpian2003@scau.edu.cn (R.P.); 2 Root Biology Center, South China Agricultural University, Guangzhou 510642, China; 3 College of Natural Resources and Environment, South China Agricultural University, Guangzhou 510642, China; 4 Key Laboratory of Agricultural and Rural Pollution Control and Environmental Safety in Guangdong Province, Guangzhou 510642, China

**Keywords:** cadmium transporter, cadmium toxicity, phytoremediation, cadmium-related genes, cadmium transcription factor

## Abstract

Cadmium (Cd) exerts a toxic influence on numerous crucial growth and development processes in plants, notably affecting seed germination rate, transpiration rate, chlorophyll content, and biomass. While considerable advances in Cd uptake and detoxification of plants have been made, the mechanisms by which plants adapt to and tolerate Cd toxicity remain elusive. This review focuses on the relationship between Cd and plants and the prospects for phytoremediation of Cd pollution. We highlight the following issues: (1) the present state of Cd pollution and its associated hazards, encompassing the sources and distribution of Cd and the risks posed to human health; (2) the mechanisms underlying the uptake and transport of Cd, including the physiological processes associated with the uptake, translocation, and detoxification of Cd, as well as the pertinent gene families implicated in these processes; (3) the detrimental effects of Cd on plants and the mechanisms of detoxification, such as the activation of resistance genes, root chelation, vacuolar compartmentalization, the activation of antioxidant systems and the generation of non-enzymatic antioxidants; (4) the practical application of phytoremediation and the impact of incorporating exogenous substances on the Cd tolerance of plants.

## 1. Introduction

It is well known that the rapid evolution of modernized industrial and agricultural practices has led to various environmental challenges. One of the prominent problems is cadmium (Cd) pollution. Between 1950 and 1990, global cadmium production doubled, reaching approximately 20,000 tons annually. Anthropogenic sources deposited an estimated 900 to 3600 tons of Cd into aquatic environments [1], resulting in severe Cd pollution. This pollution poses a significant threat to both human health and environmental safety.

Cd pollution is a global concern, evident in various regions. In the suburbs of Dera Ismail Khan, Pakistan, vegetables cultivated using wastewater irrigation exhibited significantly higher levels of Cd accumulation compared to those grown with freshwater irrigation [2]. In southern China, tobacco–rice rotation causes soil pH to decrease, thereby enhancing the flow of cadmium to crops [3]. Notably, the transfer of cadmium from the soil to the human body through crops such as vegetables and rice can lead to a variety of health problems, such as central nervous system depression and kidney and liver damage [4]. In the last century, many people in Japan suffered immensely from Itai-itai disease due to cadmium contamination of farmland and water sources [4].

To cope with these formidable challenges, numerous scientists have dedicated their efforts to environmental science research, trying to explore strategies for mitigating Cd pollution through a variety of pathways, including physical, chemical, and phytoremediation. Based on previous studies, this review will focus on the mechanisms of Cd transport by plants, the forms of Cd toxicity suffered/coped by plants, and the effects of exogenous modes on phytoremediation.

## 2. The Pollution Status and Hazards of Cadmium

Cd is a rare dispersed element commonly found in soils and zinc (Zn) minerals in the form of divalent cations (Cd^2+^) and soluble complexes with a variety of toxic effects. Cd has an extremely long biological half-life, predominantly accumulating in the liver and kidneys of the human body where it is difficult to eliminate [5].

### 2.1. Sources and Distribution of Cadmium

Cd levels vary in different countries around the world due to geographic location, latitude and longitude, and environmental climate. Cd levels in soils are currently higher than the original environmental background values in all countries as a result of atmospheric deposition and overuse of phosphate fertilizers. For example, the average concentration of Cd in European soils is 0.33 mg kg^−1^ [6], while in agricultural land in China, it is 0.19 mg kg^−1^, with an environmental background value of 0.097 mg kg^−1^ [7]. In the United States agricultural soils, the average Cd concentration is 0.265 mg kg^−1^ [8].

**Figure 1:**
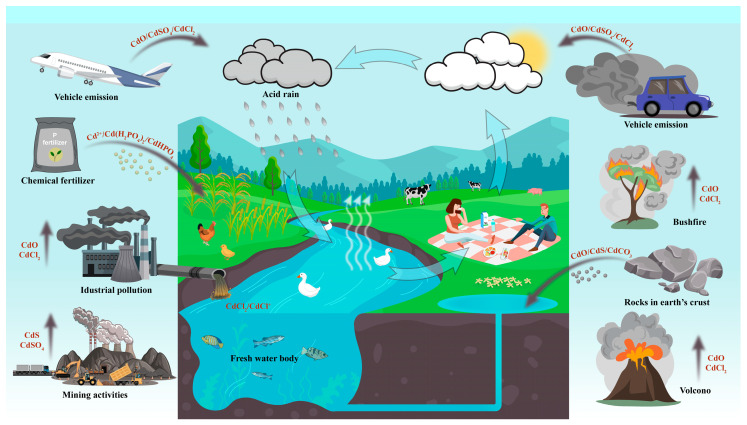
Sources of Cd and its transmission pathways in the environment. This figure shows the process of Cd deposition, accumulation, cycling in the environment, and enrichment in plants, animals, and humans through the food chain. Cd exists in different chemical forms in each pathway, such as in water bodies, where it is mainly in the form of chloride [15], in coal combustion and dust formed in non-ferrous metal production, where it mainly contains CdS and CdSO_4_ [16], and in phosphorus fertilizers in the form of Cd(H_2_PO_4_)_2_ and CdHPO_4_ [17].

Soil Cd comes from a wide range of sources and can be categorized into two main sources, including natural and anthropogenic sources. The presence of Cd in natural soils mostly originates from rock weathering and suspended soil particles transported through the air. Soil particle sources encompass various natural occurrences such as forest fires, volcanic emissions, and atmospheric dust [9]. In contrast, anthropogenic Cd emissions predominantly originate from activities like phosphate fertilizer application, tailings disposal, metal industry practices, mining operations, and fossil fuel combustion [10,11]. Since the Industrial Revolution, diverse industrial, mining, and agricultural activities worldwide have led to substantial heavy metal diffusion into soils and water bodies (Figure 1). By the beginning of the 21st century, global anthropogenic Cd production during the industrial era had accumulated to 1.1 million tons, with the global per capita burden estimated at 0.18 kg [12].

### 2.2. Hazards of Cadmium on the Human Body

With the escalation of mining activities and metal smelting, there has been a corresponding increase in Cd levels found in soil surfaces, air, and water sources. This raises a significant hazard to the health of animals, plants, and human beings. Cd in animals and humans mainly comes from drinking water, eating, and respiration, and a very small part is absorbed through the skin and hair. When Cd enters the body through the gastrointestinal or respiratory tract, it is transported into the bloodstream through erythrocytes and albumin, and then accumulates in the kidneys and liver [5]. Of note, the biological half-life of Cd in the kidney is 45 years [13].

To minimize the potential harm caused by exposure to or inhalation of Cd, individuals should consume foods that are rich in polyphenols [14], such as mint and strawberries. These foods possess antioxidant properties and can aid in chelating Cd^2+^. Additionally, incorporating more seafood, legume products, melon seeds, and other foods with high Zn content into one’s diet can help counteract the excessive accumulation of Cd in the body. Collectively, these dietary measures operate through distinct mechanisms to support overall physical well-being.

## 3. Mechanisms of Cadmium Uptake and Transport in Plants

### 3.1. Forms and Bioavailability of Cadmium in Soils

The chemical forms of Cd in different soils vary depending on conditions such as soil redox potential, moisture, texture, and inter-root environment. According to the five-step sequential extraction method proposed by Tessier et al. (1979), Cd in soils can be grouped into five forms: soluble and exchangeable state (Cd^2+^), carbonate-bound state (CdCO_3_), ferromanganese-oxidized state, organic-bound state, and residue [18], with soluble and exchangeable states dominating.

Not all Cd in soils can be absorbed by plants. Plants mainly take up the exchangeable and carbonate-bound states of Cd. However, the bioavailability of Cd can also be influenced by the soil’s properties. Among them, soil acidity and alkalinity will significantly affect the Cd solubility and morphological distribution in soils, and increasing soil pH is negatively correlated with the effectiveness of heavy metals in plants [19,20]. The main reason for the increased Cd pollution in recent years is soil acidification. In addition, soil properties are also a key factor influencing Cd morphology and utilization, and plants in sandy soils are more capable of Cd uptake compared with clay soils [21]. It is worth mentioning that the chelating agents such as ethylenediaminetetraacetic acid (EDTA) and nitrilotriacetic acid (NTA) can increase the effectiveness of Cd in soils, facilitate its movement from roots to aboveground tissues, and increase Cd accumulation in leaves [22]. In addition to Cd bioavailability, Cd uptake and accumulation in plants are also influenced by other factors, such as plant species, genotype, inter-root environment, and mineral nutrients such as silicon (Si), selenium (Se), and iron (Fe) [23,24,25,26].

### 3.2. Mechanism of Cadmium Uptake by Plants

**Figure 2:**
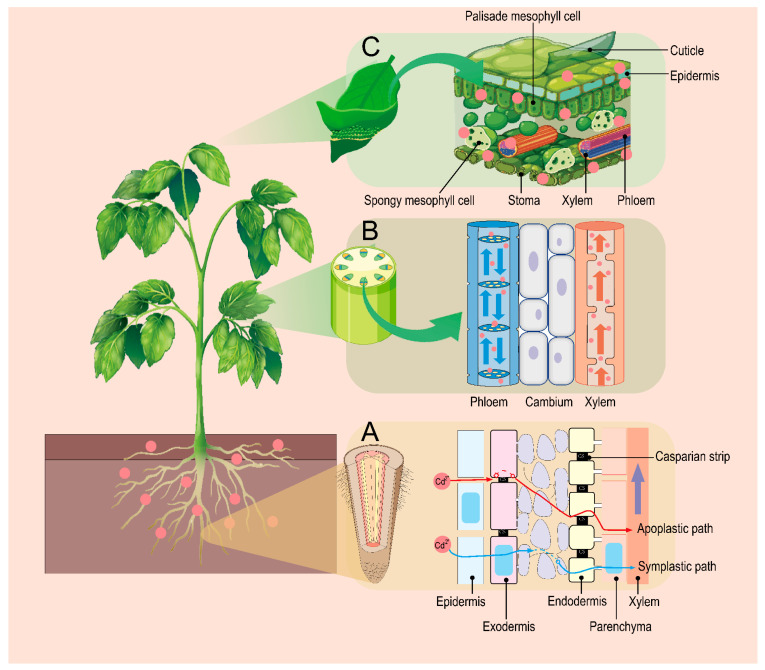
Pathways of Cd uptake and transfer by plants through roots, stems, and leaves. (**A**) The pathway of Cd uptake in plant roots from root hair cells in the maturation zone of the root tip, through the exodermis and endodermis to the stele (Xylem). (**B**) The translocation process of Cd to the aboveground parts through the xylem and phloem after reaching the stele. (**C**) Schematic diagram of Cd uptake and transport by plant leaves under Cd stress.

Cd uptake and accumulation in plants mainly consist of the following physiological processes: uptake of Cd from the outside (soil or air), lateral and radial transport of Cd, and phloem-mediated aboveground redistribution (Figure 2).

#### 3.2.1. Pathways of Cadmium Transport from Soil to Root Epidermis

The root is the first organ exposed to Cd in soils and the first barrier for plants to resist Cd toxicity. Plants absorb Cd from the soil through root hairs and epidermal cells in the mature zone of the root tip.

The entry of Cd into roots mainly includes the following pathways:

1. Cd^2+^ is exchanged with H^+^ produced during plant respiration and is thus adsorbed on the surface of root epidermal cells and then enters the cortex via the apoplast pathway [27].
2. As Cd is a non-essential metallic element lacking a specific transporter in plants, its entry into plant tissues typically occurs through the symplast pathway. This process involves competing for binding sites on metal transporter proteins, including IRT1 (a bivalent iron transporter protein) and NRAMP5 (a manganese transporter protein) [28]. Additionally, Cd can also enter plants through ion channels of divalent metals such as calcium (Ca).
3. To enhance ion utilization within the inter-root soil, certain low molecular compounds, such as erucic acid and oxalic acid, are secreted by plant roots. These compounds form metal–ligand complexes with Cd^2+^, thus facilitating Cd to enter the root epidermis as a chelate transported by yellow-stripe-Like (YSL) proteins [29].

Although Cd^2+^ binds to both organic ligands (such as dissolved organic substances like low-molecular-weight organic acids secreted by plant roots) and inorganic ligands (e.g., NO^3−^, Cl^−^) to form soluble complexes, subsequently enhancing the mobility of Cd to the root surface, it is widely accepted that Cd^2+^ is the primary form of translocation through the plasma membrane into the root tip cells [30].

#### 3.2.2. Lateral Transport of Cadmium from Root Epidermis to Xylem

Cd is synergistically transported through both the symplast and the apoplast pathways after entering the epidermal cells of the root hair zone (Figure 2).

In the symplast pathway, Cd is mainly transported by intracellular protoplasmic flow and plasmodesmata channels between cells, first across the endodermis into the stele, then through the pericycle sheaths into the parenchyma cell, and ultimately into the xylem conductance ducts [31]. In the apoplast pathway, Cd enters the xylem along the apoplast space such as the cell wall, cell interstitial space, intercellular layer, and conduit cavity. This pathway is impeded by casparian strips through the endodermis. Additionally, a fraction of the Cd^2+^ is sequestered into vacuoles through transporter proteins like OsHMA3 in rice [32], redirected to the symplast pathway for Cd transportation to the xylem of the stele [33].

Cd transport from the epidermis to the xylem completes within the root, and the entire mechanism is mediated by transporter proteins in the plasma membrane, which directs the ions to specific locations [34].

#### 3.2.3. Radial Transport of Cadmium through the Xylem to Aboveground Parts

Upon Cd entering into roots, the xylem pathway subsequently plays a dominant role in the long-distance transport of Cd from roots to aboveground (Figure 2), with the main driving forces being transpiration pull and root pressure [35].

It is known that Cd accumulates mainly in the roots of plants and is partially translocated from roots to aboveground parts. The efficiency of translocation depends on root vacuole sequestration and xylem loading capacity [30]. The more vacuole sequestration in the root, the less Cd^2+^ is transferred to the aboveground part. As documented, the transporter proteins CAX2, CAX4, and HMA3 play key roles in chelating Cd to the vacuole [32,36,37]. In addition, the lignified casparian strips in the endodermis of plant roots serve as a barrier, impeding the entry of Cd into the shoots. This may account for the higher accumulation of Cd in the roots in comparison to the shoots [38].

#### 3.2.4. Phloem Mediates Cadmium Redistribution

The phloem is mainly responsible for the redistribution of Cd in the aboveground part based on studies in *Arabidopsis thaliana* [39] and rice [40].

Cd absorbed by plant roots is mainly translocated to the aboveground part through the xylem and then to the rice grain through the phloem [41], suggesting that most of the Cd needs to be translocated from the xylem to the phloem before it can be re-translocated to the grain. In recent years, new insights into the process of Cd translocation from roots to stems have been gained, suggesting that past studies have overlooked the value of long-distance transport in the phloem [42]. It was found that the phloem is an important pathway for Cd long-distance transport from root to leaf in eggplant and oilseed rape [35,43], and that Cd-GS_2_ complexes contribute to Cd long-distance movement in the phloem [42].

### 3.3. Cadmium-Related Transporter Proteins in Plants

**Figure 3:**
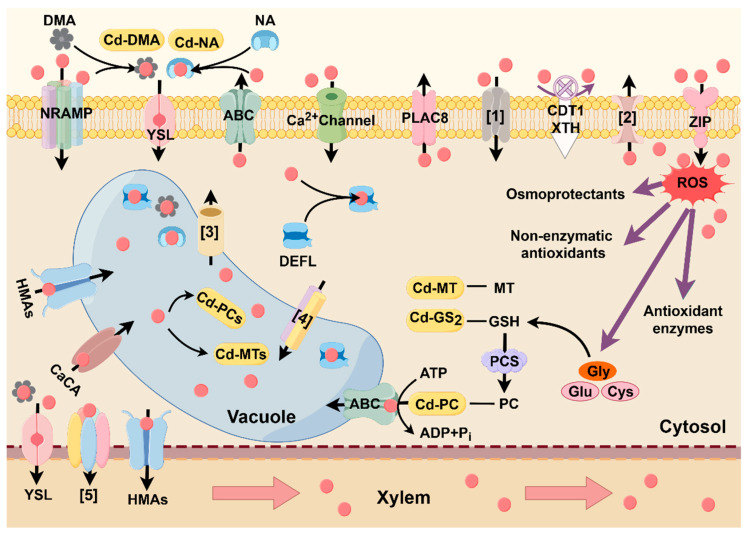
Schematic model of the major proteins/enzymes that are absorbed, transported, sequestered, and detoxified in plants. Red circles represent Cd^2+^ and [number] represents the serial number. Plants take up Cd and Cd chelates through NRAMP, YSL, ZIP families, and Ca^2+^ channels; ABC and PLAC8 families have been shown to function in effluxing Cd out of the plant; CDT1 and XTH can avoid Cd entry by binding Cd or by reducing the Cd-binding site; the DEFL family can bind to Cd and convert Cd ions into stable compounds; HMA, CaCA, and ABC families can transport Cd and chelates into vacuoles to alleviate the toxic effects; YSL and HMA can transport some Cd to xylem and transfer it to the aboveground part. Refs. [1,2,3,4,5] are proteins that have been reported to be related to Cd transport. Ref. [1] SpHMA6, SaPCR2, SlCNR8, PcPLAC8-10, OsCd1, OsHIR1, OsAAN4, and OsGLR3.4, respectively; Ref. [2] SlCNR8, OsZIP1, OsHMA9, HaMTP10, AtPDF2.5, OsCCX2 (OsCDT1), OsLCT1; Ref. [3] OsNRAMP2, AtNRAMP3, AtNRAMP4; Ref. [4] TmMTP1, TmMTP11, AtCAX2; Ref. [5] OsZIP7, OsCAL1. On the right is the Cd-induced ROS scavenging cycle. Cd enters the cytoplasm and stimulates the synthesis of osmoprotectants, antioxidants, glutathione and phytochelatin, and metallothionein. MT, GSH, and PC can bind to Cd to generate Cd-GS_2_, Cd-MT, and Cd-PC to alleviate the toxicity of Cd caused to the cells. ROS, reactive oxygen species; NRAMP, natural resistance-associated macrophage protein; YSL, yellow-stripe-1-like; ABC, ATP-binding cassette family; PLAC8, the placenta-specific 8-domain-containing family; ZIP, ZRT-IRT-like protein family; zinc-regulated; HMA, heavy metal ATPase; CaCA, cation/calcium superfamily; DEFL, defensin-like protein family; PCS, phytochelatin synthetase; Gly, Glycine; Glu, Glutamate; Cys, Cysteine; MT, metallothioneins; GSH, glutathione; PC, phytochelatin; NA, nicotianamine; DMA,2’-deoxymugineic acid.

Cd is one of the non-essential metal ions; the uptake of Cd by plants from soils must be mediated by transporter proteins for essential cations [44,45]. As shown in Figure 3 and Table 1, the following protein families are commonly involved in Cd transport or detoxification.

#### 3.3.1. The Natural Resistance-Associated Macrophage Protein Family

The natural resistance-associated macrophage protein (NRAMP) family has been identified in model plants such as *Arabidopsis thaliana* and rice. As revealed, NRAMP plays a key role in metal homeostasis.

A total of seven NRAMP family members in rice have been functionally characterized. NRAMP1, NRAMP2, and NRAMP5 are involved in Cd transport [46]. OsNRAMP2 is localized to the tonoplast and is primarily expressed in seeds, roots, and leaves [47]. Knockout of *OsNRAMP2* resulted in a reduction in Cd translocation from vegetative tissues to rice grains; conversely, overexpression lines of *OsNRAMP2* exhibited a significant increase in grain Cd concentrations [48,49]. OsNRAMP5 is localized in the plasma membrane and expressed in rice roots [50]. OsNRAMP5 serves as the primary transporter protein for manganese (Mn) and Cd, facilitating their translocation from soil solution to root cells. The knockout lines of *OsNRAMP5* have a substantial reduction in Cd in rice, and overexpressing *OsNRAMP5* increases Cd uptake in roots, but Cd levels in shoots remain at low levels [51,52]. OsNRAMP1 is localized in root and leaf cells and is a close homolog to OsNRAMP5 (72.78% amino acid sequence similarity) with similar but non-redundant functions [53,54,55]. Knockout of *OsNRAMP1* or *OsNRAMP5* results in reduced levels of Cd in rice. Moreover, the loss of function of *OsNRAMP5* has a greater impact than *OsNRAMP1*. Further studies have revealed that double knockout mutants of these two genes have a significant reduction in Cd [53]. This indicates that gene editing of *OsNRAMP1* or/and *OsNRAMP5* can help in the breeding of low-Cd varieties of rice, especially by altering their expression levels via editing the promoter sequence in the future. It must be pointed out that the uptake of Mn is also impaired by the knockout of these two genes. As Mn is one of the essential elements for plant growth and development, how to balance the relationship between the two elements is an important goal for future investigations.

#### 3.3.2. The Zinc/Iron-Regulated Transporter-like Protein Family

Lots of studies have demonstrated that the zinc–iron-regulated transport proteins (ZIPs) family plays an important role in metal uptake in roots and distribution in plants.

To date, 15 ZIP family members have been reported in *Arabidopsis thaliana* [56]. *AtIRT1*, the first identified ZIP family member in *Arabidopsis thaliana*, is mainly expressed in roots and plays a key role in the uptake of divalent iron from the soil [57,58]. Moreover, AtIRT1 is involved in the transport of divalent cations such as Zn, Mn, cobalt (Co), and Cd [58,59,60,61]. Knockout of *AtIRT1* results in lower accumulation of the heavy metal Cd [60]. Although AtIRT2 shares phylogenetic similarity with AtIRT1, it is not directly responsible for Fe uptake from soils. Instead, AtIRT2 collaborates with AtIRT1 to maintain Fe homeostasis in plants [62,63]. Overexpression of *AtIRT2* in *Arabidopsis thaliana* enhances the uptake of metals such as Fe, Cd, and Zn. However, the sensitivity of yeast cells to Cd remains unaffected when overexpressing *AtIRT2* [62], possibly due to the indirect synergistic interaction between AtIRT2 and AtIRT1 in response to Cd stress. AtZIP1 is a vacuole transporter that transfers Mn [64]. AtZIP2 participates in the uptake of Mn and Zn [64]. Interestingly, *AtZIP1*, *AtZIP3*, and *AtZIP4* respond to Zn deficiency [65]. In addition, AtZIP2 and AtZIP4 are also involved in copper (Cu) transport [66]. In short, ZIP family members have versatile roles in Mn, Zn, and Cd transportation.

There are 16 ZIP family members in rice [67]. OsIRT1 and OsIRT2 are mainly responsible for the uptake of Zn and Fe in the rice root system [68] and also play a role in Cd uptake because of the similar physicochemical properties of Zn^2+^, Fe^2+^, and Cd^2+^ [69,70]. OsZIP1*,* localized in the endoplasmic reticulum and plasma membrane, is a metal detoxification transporter. Overexpressing *OsZIP1* can reduce the overaccumulation of Zn, Cu, and Cd in rice and promote rice growth [71,72]. OsZIP3 is preferred for Zn uptake over other divalent cations such as Cd. OsZIP3 co-regulates Cd transport and uptake together with OsHMA2 and OsLCT1 [73,74]. Both *OsZIP5* and *OsZIP9* are redundantly involved in Zn and Cd uptake. *OsZIP9* is responsible for the broad regulation of Zn in roots and shoots, and *OsZIP5* fine-tunes Zn uptake to maintain Zn homeostasis. Accordingly, rice with a single or both genes knocked out exhibits reduced uptake of Zn and Cd, whereas overexpression of *OsZIP5* or *OsZIP9* has the opposite effect [75]. OsZIP6 demonstrates transport activity for Fe^2+^, Cd^2+^, and Co^2+^, exhibiting the highest efficiency under acidic environmental conditions [76]. *OsZIP7* is expressed in parenchyma cells of vascular bundles in rice roots and nodes and is involved in the transport of Zn and Cd. In line with this, the Cd levels in the roots and internode of knockout lines of *OsZIP7* are higher [77].

Different species have different expression patterns in Cd stress, *Arabidopsis thaliana* up-regulates ZIP family genes in the roots, and rice mainly up-regulates aboveground ZIP family genes. It has been verified that AtIRT1, OsZIP1, and OsZIP3 play more important roles in Cd uptake [78].

#### 3.3.3. The Heavy Metal ATPases

Known as P_1B_-ATPase, heavy metal ATPase (HMAs) plays an important role in the translocation or detoxification of heavy metals in plants [79], especially in hyperaccumulators. These transporters are reported to exhibit variations in various aspects, including subcellular localization and metal specificity. When plants are exposed to low concentrations of Cd stress, only a limited number of *HMA* genes are up-regulated. The majority of transporters are mobilized only in response to elevated concentrations [80].

The rice genome encodes nine heavy metal ATPases. OsHMA1 and OsHMA4 have higher expression levels when stressed with Cd. Generally, OsHMA1 and OsHMA4 play roles in maintaining homeostasis in plants under heavy metal stress [80], but their specific biological functions remain to be explored. OsHMA2 is localized in the plasma membrane of the root stele. OsHMA2 is mainly involved in mediating the xylem loading of Cd and Zn, and the translocation to the shoots [69,81]. *OsHMA3* is expressed in the vesicular membrane of root cells and is responsible for chelating extra-membranous Cd and Zn to the vacuole to prevent their translocation to the aboveground organs [32,45]. *OsHMA9* is expressed in root and mesophyll tissue and appears to be responsible for Cd, Cu, Zn, and Pb efflux [82].

*Arabidopsis thaliana* genome encodes eight HMAs. AtHMA1 is localized in the chloroplast periplasm and has been found to transport not only Cu and Zn [83,84], but also Cd and Ca after heterologous expression in yeast [85]. AtHMA2 and AtHMA4 are localized in the plasma membrane and mediate the translocation of Cd from roots to shoots [86,87,88,89]. AtHMA3 showed similar properties to OsHMA3 and mediated the vacuole sequestration of Cd in roots. Consistently, overexpression of *AtHMA3* results in enhanced tolerance of *Arabidopsis thaliana* to Zn, Co, Cd, and Pb [90].

In summary, the heavy metal ATPase family can be divided into two groups based on the properties of the metal substrates; the first group is the Zn/Co/Cd/Pb subgroup as exemplified by rice *OsHMA1–OsHMA3* and *Arabidopsis thaliana AtHMA1–AtHMA4*, and the second is the Cu/Ag subgroup, containing rice *OsHMA4–OsHMA9*, *Arabidopsis thaliana AtHMA5–AtHMA8* [91]. As the second group of HMA is not related to Cd, we do not introduce them here.

#### 3.3.4. Others

In addition to the above family of transporter proteins, there are many other transporters also involved in the uptake, transport, and efflux of Cd (Table S1).

In rice, the common ones are Cd Accumulation in Leaves 1 (*OsCAL1*), Plant Cd Resistance 1 (*OsPCR1*), Low Cd (*OsLCD*), Low-affinity Cation Transporter genes 1 (*OSLCT1*), Cd transporter genes 1 (*OsCd1*) and Hypersensitive Induced Reaction Protein 1 (OsHIR1). OsCAL1 is responsible for chelating Cd and exports it from the cytoplasm to the outside of the cell, thus reducing Cd concentration in the cells [92,93]. OsPCR1 is involved in the transport of rice from roots to aerial parts [94]. *OsLCD* is mainly expressed in roots and leaves, and its absence reduces the accumulation of Cd in plants [95]. OsLCT1 is localized at the plasma membrane and exhibits Cd efflux activity in yeast [96,97]. As reported, knockout lines of *OsLCT1* have lower Cd levels in phloem and grains [96,97]. OsCd1 belongs to the Major Facilitator Superfamily (MFS) family of transporter proteins. OsCd1 is localized in the plasma membrane of roots. OsCd1 is associated with Cd uptake in roots and contributes to Cd accumulation in rice grains [93,98]. Heterogeneous overexpressing *OsHIR1* significantly reduces Cd and arsenic (As) accumulation, thus increasing plant tolerance to Cd and As [99].

**Table 1:** Gene family related to Cd uptake, transport and efflux.

Gene Family	Plant	Gene	Expression Site	Function	Reference
The natural resistance-associated macrophage protein family (NRAMP)	*Oryza sativa* L.	*OsNRAMP1*	Root cells and leaf mesophyll cells	Cd uptake and transport	[53,54,55]
		*OsNRAMP2*	Seeds, roots, leaf sheaths and leaf blades	Cd efflux, translocation and distribution	[47,48,49]
		*OsNRAMP5*	Roots	Cd uptake	[28,50,51]
	*Arabidopsis thaliana*	*AtNRAMP1*	Roots and aerial parts	Cd entry and transport	[100,101]
		*AtNRAMP3*	Roots and aerial parts	Cd transport	[100,102,103]
		*AtNRAMP4*	Roots and aerial parts	Cd transport	[103,104]
		*AtNRAMP6*	Seeds and shoots	Cd transport and distribution	[105]
	*Nicotiana tabacum* L.	*NtNRAMP1*	Roots	Cd uptake and accumulation	[106]
		*NtNRAMP3a*	Leaves	Cd transport, tolerance and accumulation	[107]
		*NtNRAMP3b*	Leaves and roots	Cd uptake, transport and maintain homeostasis	[108]
		*NtNRAMP5*	Roots	Cd transport	[109]
		*NtNRAMP6a*	Roots, stems, leaves and flowers	Cd transport	[110]
		*NtNRAMP6b*	Roots, stems, leaves and flowers	Cd transport	[110]
The natural resistance-associated macrophage protein family (NRAMP)	*Sedum alfredii* Hance	*SaNRAMP1*	Shoots	Cd transport and accumulation	[111]
		*SaNRAMP3*	vascular tissues	Cd transport	[112]
		*SaNRAMP6*	Leaves and roots	Cd transport and accumulation	[113]
	*Sedum plumbizincicola*	*SpNRAMP5*	-	Cd transport	[114]
	*Populus × canescens*	*PcNRAMP1*	Roots	Cd uptake and transport	[115]
	*Morus alba*	*MaNRAMP1*	Roots	Cd transport	[116]
	*Populus trichocarpa*	*PtNRAMP1*	Leaves and roots	Cd transport	[117]
		*PtNRAMP2*	Leaves and roots	Cd transport	[117]
		*PtNRAMP4*	Leaves and roots	Cd transport	[117]
		*PtNRAMP9*	Leaves and roots	Cd transport	[117]
		*PtNRAMP10*	Leaves and roots	Cd transport	[117]
		*PtNRAMP11*	Leaves and roots	Cd transport	[117]
	*Malus hupehensis*	*MhNRAMP1*	Roots	Cd uptake and accumulation	[118]
	*Malus baccata* (L.) Borkh	*MbNRAMP1*	Roots	Cd transport	[119]
	*Noccaea caerulescens* (*Thlaspi caerulescens*)	*NcNRAMP1*	Roots	Cd transport	[120]
		*TcNRAMP3*	Roots	Cd accumulation and homeostasis	[121,122]
		*TcNRAMP4*	Roots	Cd transport	[122]
The natural resistance-associated macrophage protein family (NRAMP)	*Brassica rapa* L*. Chinensis.*	*BcNRAMP1*	Whole plant body	Cd uptake and accumulation	[123]
	*Brassica napus*	*BnNRAMP1b*	Seedlings and vegetative tissue	Cd transport	[124]
	*Triticum polonicum* L.	*TpNRAMP5*	Roots and basal stems	Cd transport	[125]
	*Hordeum vulgare*	*HvNRAMP5*	Roots	Cd uptake	[126]
	*Spirodela polyrhiza*	*SpNRAMP1*	Roots, fronds and joint between mother and daughter fronds	Cd uptake and accumulation	[127,128]
	*Vigna radiata*	*VrNRAMP5*	Roots	Cd uptake	[129]
The zinc/iron-regulated transporter-like protein family (ZIP)	*Oryza sativa* L.	*OsIRT1*	Roots	Cd uptake and transport	[68,70,130]
		*OsIRT2*	Roots	Cd uptake	[130,131]
		*OsZIP1*	Roots	Cd efflux	[71,73]
		*OsZIP3*	Nodes	Cd transport and distribution	[73,74,132]
		*OsZIP5*	Roots	Cd uptake	[75]
		*OsZIP6*	Shoots and roots	Cd transport	[76]
		*OsZIP7*	Roots and nodes	Cd transport	[77]
		*OsZIP9*	Shoots and roots	Cd uptake	[75]
	*Arabidopsis thaliana*	*AtIRT1*	Roots	Cd uptake and transport	[58,59,60,133]
The zinc/iron-regulated transporter-like protein family (ZIP)	*Nicotiana tabacum* L.	*NtIRT1*	Roots	Cd uptake and accumulation	[134,135]
		*NtZIP1* *(NtZIP5B)*	Roots	Cd uptake	[136,137]
		*NtZIP4B*	Leaves and roots	Cd transport	[137]
	*Arabidopsis halleri*	*AhZIP6*	Leaves and roots	Cd transport, tolerance	[138]
	*Noccaea* caerulescens (*Thlaspi caerulescens*)	*TcZNT1*	Roots	Cd uptake	[139]
		*TcZNT5*	Roots	Cd transport	[140]
		*TcZNT6*	Shoots and roots	Cd transport	[140]
	*Sedum alfredii* Hance	*SaZIP4h*	Shoots and roots	Cd transport	[141]
	*Morus alba*	*MaIRT1*	Leaves	Cd transport	[116]
		*MaZIP4*	Roots	Cd transport	[116]
	*Thlaspi japonicum*	*TjZNT1*	-	Cd transport	[142]
		*TjZNT2*	-	Cd transport	[142,143]
	*Brassica chinensis* L.	*BcIRT1*	-	Cd transport	[144]
		*BcZIP2*	-	Cd transport	[144]
	*Avicennia marina*	*AmZIP1*	Roots	Cd transport	[145]
		*AmIRT1*	Leaves, stems, and roots	Cd transport	[145]
	*Triticum polonicum* L.	*TpIRT1A/B*	Roots, leaves, and reproductive organs	Cd uptake and transport	[146]
The heavy metal ATPases (The P_1B_-type ATPases family)	*Oryza sativa* L.	*OsHMA9*	Roots and mesophyll tissues	Cd efflux	[82]
	*Arabidopsis thaliana*	*AtHMA2*	Vascular tissues of roots, stems, and leaves	Cd transport and homeostasis	[86,87]
	*Arabidopsis halleri*	*AhHMA4*	Shoots and roots	Cd transport	[147]
	*Sedum alfredii* Hance	*SaHMA3h*	Shoots and roots	Cd transport and sequestration within vacuoles	[148]
		*SaHMA3n*	Shoots and roots	Cd transport and sequestration within vacuoles	[148]
	*Sedum plumbizincicola*	*SpHMA6*	Leaves and roots	Cd uptake, translocation and distribution	[149]
	*Noccaea caerulescens* (*Thlaspi caerulescens*)	*TcHMA4*	Shoots and roots	Cd transport	[150]
	*Morus alba*	*MaHMA3*	Roots	Cd transport	[116]
	*Capsicum* sp.	*CaHMA1*	Pepper fruits	Cd transport and accumulation	[151]
	*Glycine Max* (L.) Merr.	*GmHMA3w*	Roots	Cd transport and sequestration within endoplasmic reticulum	[152]
	*Triticum aestivum* L.	*TaHMA2*	-	Cd translocation and transport	[153]
	*Avicennia marina*	*AmHMA2*	Roots, leaves, stems, buds, and flowers	Cd transport	[145]
The yellow-stripe-like transporter (YSL)	*Solanum nigrum*	*SnYSL3*	Roots and stems	Cd-NA compound transport	[154]
	*Brassica juncea*	*BjYSL7*	Stems	Cd transport and tolerance	[155]
	*Zea mays* L.	*ZmYS1*	-	Cd-DMA compound transport	[156]
	*Vaccinium* ssp.	*VcYSL6*	-	Cd-NA compound transport	[157]
The ATP-binding cassette transporter family (ABC)	*Oryza sativa* L.	*OsABCG36* *(OsPDR9)*	Roots and shoots	efflux of Cd and Cd chelates	[158]
		*OsABCG43* *(OsPDR5)*	Roots and shoots	Cd transport and tolerance	[159]
		*OsPDR20* *(OsABCG53)*	Whole plant body	efflux of Cd and Cd chelates	[160]
	*Arabidopsis thaliana*	*AtPDR8* *(AtABCG36)*	Roots	Cd efflux	[161]
		*AtATM3* *(AtABCB25)*	Roots	Cd chelates transport	[162,163]
	*Populus tomentosa*	*PtoABCG36*	Leaves, stems and roots	Cd efflux	[164]
	*Sedum plumbizincicola*	*SpABCB28*	-	Cd transport into organelles	[114]
	*Rehmannia glutinosa*	*RgABCC1*	Roots	Cd transport	[165]
The placenta-specific 8-domain -containing family (PLAC8)	*Oryza sativa* L.	*OsPCR1* *(OsFWL5)*	Grains, roots, stems and leaves	Cd accumulation and transport	[166]
		*OsPCR3* *(OsFWL2)*	Grains, roots, stems and leaves	Cd accumulation and transport	[166,167]
		*OsFWL3*	-	Cd tolerance	[168]
		*OsFWL4*	-	Cd transport and translocation	[168]
	*Populus euphratica*	*PePCR2*	Roots	Cd efflux	[169]
		*PePCR10*	-	Cd efflux	[170]
	*Sedum alfredii* Hance	*SaPCR2*	Roots	Cd uptake and accumulation	[171]
	*Brassica napus*	*BnPCR10.1*	Whole plant body	Cd transport	[172]
	*Avicennia marina*	*AmPCR2*	Stems, pneumatophores and roots	Cd efflux	[145]
	*Salix linearistipularis*	*SlPCR6*	Roots	Cd transport	[173]
		*SlCNR8*	Roots	Cd uptake, efflux and accumulation	[174]
	*Triticum aestivum*	*TaCNR2*	Leaves and internodes	Cd transport and tolerance	[175]
	*Triticum urartu*	*TuCNR10*	Shoots and roots	Cd transport	[176]
	*Populus × canescens*	*PcPLAC8-10*	Roots	Cd uptake	[177]
The metal tolerance protein family (MTPs)	*Oryza sativa* L.	*OsMTP1* *(OZT1)*	Roots, seeds and leaves	Cd transport	[178,179,180]
	*Helianthus annuus* L.	*HaMTP10*	-	Cd efflux	[181]
The defensin-like protein family (DEFL)	*Oryza sativa* L.	*OsCAL1*	Roots	Cd chelation and transport	[92,93]
	*Arabidopsis thaliana*	*AtPDF2.5*	Roots	Cd chelation and efflux	[182]
The cation/calcium superfamily (CaCA)	*Oryza sativa* L.	*OsCAX1a*	Roots	Cd transport and tolerance	[183]
		*OsCAX1c*	Roots and leaves	Cd transport and tolerance	[183]
		*OsCAX4*	Roots and leaves	Cd transport and tolerance	[183]
The cysteine-rich peptide family (CYSTM)	*Oryza sativa* L.	*OsCCX2* *(OsCDT1)*	Nodes	Cd efflux	[184,185,186]
The low-affinity cation transporter family (LCT)	*Oryza sativa* L.	*OsLCT1*	Leaves and nodes	Cd efflux and transport	[97,187]
		*OsLCT2*	Roots	Cd transport	[188]
The major facilitator superfamily (MFS)	*Oryza sativa* L.	*OsCd1*	Roots	Cd uptake	[98]
The Proliferation, Ion and Death superfamily (PID)	*Oryza sativa* L.	*OsHIR1*	-	Cd uptake and tolerance	[99]
-	*Oryza sativa* L.	*OsAAN4*	-	Cd uptake	[189]
-	*Oryza sativa* L.	*OsGLR3.4*	-	Cd uptake	[189]
-	*Sorghum bicolor* L.	*SbEXPA11*	-	Cd uptake and transport	[190]

“-” means unspecified.NA, nicotianamine; DMA,2’-deoxymugineic acid.

### 3.4. Cadmium-Related Transcription Factors in Plants

Apart from transporter proteins, transcription factors (TFs) also play crucial roles in mediating plants’ responses to Cd stress. It has been reported that TF families such as WRKY, MYB, and NAC play direct or indirect roles in regulating Cd tolerance in plants [191,192,193]. This regulation is achieved through the control of Cd-related genes, activation of specific signaling pathways, or interaction with other proteins.

The WRKY family is one of the largest families of TF in plants. A total of seven WRKYs (*AtWRKY12*, *AtWRKY13*, *AtWRKY18*, *AtWRKY33*, *AtWRKY40*, *AtWRKY45*, and *AtWRKY60*) related to Cd tolerance are currently reported in *Arabidopsis thaliana* [133,191,194,195,196,197]. In particular, *AtWRKY45* facilitated the synthesis of phytochelatins by activating *AtPCS1* and *AtPCS2*, thereby enhancing Cd tolerance in *A. thaliana* [191]. Moreover, there are many other families of transcription factors (Table S2) that play important roles in the alleviation of Cd stress. *AtMYB49* regulates Cd accumulation through activation of the iron transport protein IRT1 and the abscisic acid (ABA) signaling pathway [192]. *AtbHLH38*/*AtbHLH39* increased the expression of *NAS1* and *NAS2* and reduced Cd accumulation. Collectively, these TFs alter plant sensitivity and tolerance to Cd in a manner that regulates downstream genes or modulates signaling pathways.

TFs are widely involved in plant growth and development. However, the large number of TF and the complex regulatory networks have led to the fact that fewer Cd-related TFs have been identified. Deciphering the functions and regulatory networks of various types of Cd-related TFs is an important goal, which can provide the basis for solving Cd pollution at the molecular level.

## 4. Toxicity of Cadmium and Detoxification Mechanism of Cadmium in Plants

### 4.1. Toxicity of Cadmium to Plants

**Figure 4:**
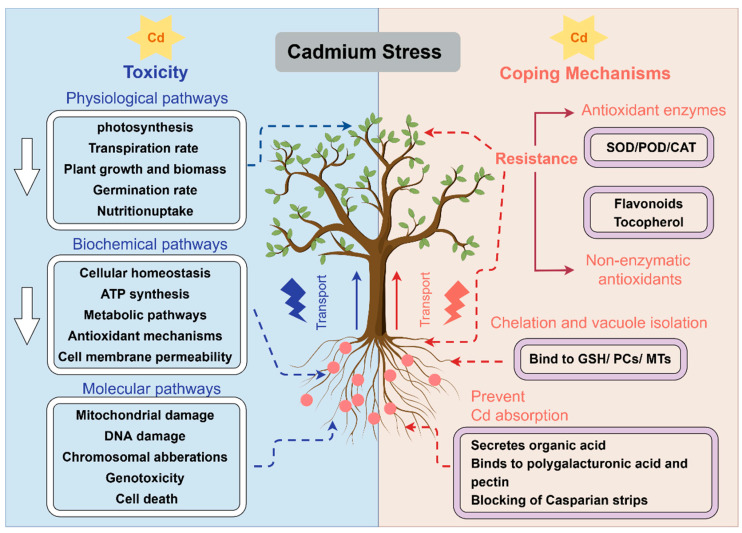
Toxic effects of Cd on plants and plant coping mechanisms. The blue part on the left shows the negative effects of Cd stress on plants, and the pink color on the right shows the mitigation mechanisms that plants have evolved over millions of years to cope with Cd stress.

Excessive Cd usually negatively affects plant growth, development, and proliferative metabolism [198], such as plant biomass accumulation, germination rate, stomatal conductance and transpiration rate (Figure 4). Potential hazards include inhibition of photosynthetic pigment formation, reduction in photosynthetic efficiency, disruption of cellular homeostasis, chromosomal aberrations, damage to mitochondria, disruption of antioxidant mechanisms and metabolic pathways, and disruption of ATP synthesis [14,199]. Of note, the proton gradient generated by the electron transport chain and photochemical reactions is the main source of ATP synthase [200]. Cd stress inhibits electron transfer during the photoresponse by acting on different sites of the PSI and PSII electron transport chain (oxygen-evolving complex on the electron donor side of PSII and sites such as Q_A_ and Q_B_ on the electron acceptor side of PSII) [201,202], thereby affecting the synthesis of plant ATPase and other physiological processes. In addition, Cd induces the production of reactive oxygen species (ROS), leading to protein oxidation, DNA damage, malondialdehyde (MDA) accumulation, and even damage to cell membranes [203]. Apart from polluting the environment and poisoning plants and animals, Cd has been reported to act as a “hormone”, i.e., at low concentrations, it activates plant defense mechanisms without causing severe oxidative stress [29,204,205]. Currently, there are few studies in this area, which need to be verified in different environments and plants.

The toxic effects of Cd are manifested in physiological and ecological aspects, but the degree of its toxic effects is related to Cd concentration and treatment time, plant species, and cultivars [206]. For instance, a low Cd concentration of 100 nM affected the growth of sunflower [207], while *Sesuvium portulacastrum* showed significant cellular damage only after 300 μM CdCl_2_ treatment [208]. Furthermore, certain plants are classified as Cd hyperaccumulators for their ability to accumulate Cd in their aerial tissues in excess of 100 mg Kg^−1^, showing high tolerance and uptake capacity [209,210].

Hence, when exploring the mechanism of plants to cope with Cd toxicity, researchers use different concentrations of Cd in hydroponic or soil culture treatments to screen and explore Cd high-tolerant plant species based on physiological data such as phenotype, plant height, root parameters, and photosynthetic efficiency, and changes in antioxidant enzyme activities.

### 4.2. Mechanisms of Plant Response to Cadmium Stress

To cope with the stress of the heavy metal Cd, plants have evolved elegant defense mechanisms (Figure 4). Firstly, when confronting Cd in soils, the plant root system secretes substances such as malic acid and citric acid, which bind to Cd^2+^ to prevent their uptake by the root system [211]. Secondly, after entering into the roots, Cd binds to polygalacturonic acid and pectin in the cell wall [212,213], thus reducing the amount of Cd in the cytosol. On the other hand, the Casparian strips on the endodermis of the root also prevent Cd from entering the cell [214]. Thirdly, chelation and vacuole isolation of Cd is also one of the important detoxification pathways. On the one hand, free Cd^2+^ binds to glutathione (GSH), phytochelatins (PCs), and metallothioneins (MTs) to form non-toxic complexes such as Cd-GS_2_, PC-Cd, MT-Cd, and so on [29]. On the other hand, plants isolate Cd^2+^ and complexes by transporting them from the cytoplasm to the vacuole through transporter proteins, thereby reducing the toxic effects of Cd on plants and enhancing their tolerance to Cd. Finally, plants keep more Cd in their roots by decreasing the long-distance transport of Cd in the xylem from root to shoot, thus mitigating the negative effects of Cd on leaves or productive organs.

When Cd crosses the barrier of plant cells, it will trigger a burst of ROS. Then the activation of antioxidant enzymes such as superoxide dismutase (SOD), peroxidase, (POD) and increased production of non-enzymatic antioxidants tocopherol and flavonoids follow (Figure 3). They are widely present in various organelles and act to eliminate the excessive accumulation of O^2-^, H_2_O_2_ and malonaldehyde to maintain intracellular environmental homeostasis. In addition, sugars, amino acids, and polyols, as an osmotic pressure regulator in plants, can maintain intracellular balance and improve plant tolerance when plants are subjected to abiotic stresses [215,216], and they can also inhibit the production of oxyradicals, scavenge excess ROS, and mitigate oxidative damage in plants [217]. It is noteworthy that salicylic acid (SA), gibberellin (GA), and ABA have been demonstrated to play pivotal roles in mitigating Cd-induced oxidative stress [29,192,218,219]. While other hormones appear to exert a regulatory influence, their specific mechanisms remain to be thoroughly explored.

According to previous studies, the above-mentioned approaches can be classified into two different strategies: avoidance and tolerance of Cd. The former is to prevent Cd from entering the cells of plants to protect plants from Cd stress, and the latter is dependent on the plant’s own tolerance and mitigation mechanisms to alleviate the negative effects of Cd. The two strategies are complementary to each other and together constitute the plant’s defense mechanism against Cd toxicity.

## 5. Effect of Exogenous Substances on Phytoremediation of Soil Cadmium Pollution

It is obvious that high levels of heavy metals negatively affect plant growth and development. Under the strong selective pressures exerted by heavy metal in soils, many plants have evolved sophisticated biological mechanisms for resisting, tolerating, or thriving in metal-bearing soils, and are collectively referred to as heavy-metal plants [220]. Of these, those that can only survive in contaminated areas are known as obligate metallophytes [221].

### 5.1. Status of Phytoremediation and Its Application

Phytoremediation is a remediation technique via plants or soil microorganisms to reduce pollutants in the surrounding environment [222], which include heavy metals, radionuclides, or organic pollutants such as polynuclear aromatic hydrocarbons (PAHs), polychlorinated biphenyls (PCBs) and pesticides in soils or air [223]. Compared with traditional physical and chemical methods, phytoremediation is green, low-cost, and highly effective [224].

Currently, the most used plants for phytoremediation of Cd-excessive soils are hyper-enriched plants, which accumulate more than 0.01 percent of Cd in their aboveground dry weight [225]. Although more than 450 plant species have been identified as metal hyper-enriched plants, only a few plant species are recognized as Cd hyper-enriched, such as *Solanum nigrum*, *Phytolacca acinosa*, and *Sedum plumbizincicola* [226]. More interestingly, these Cd-hyper-enriched plants were also Zn-hyper-enriched plants and vice versa [227]. The Cd extraction capacity of plants was different in soils, with the Cd bioconcentration factor (BCF) of plant leaves being larger in acidic soils and smaller in alkaline soils [226].

In addition, woody plants are receiving increased attention as an alternative phytoremediation technique. Recently, it has been found that fast-growing woody plants can accumulate high levels of heavy metals. For instance, a one-year-old willow can extract 17% of the Cd in soils [228], and a four-year-old *Averrhoa carambola* can remove 0.3% to 51.8% of the total Cd from soils at the surface of 20 cm [229]. This suggests that woody plants have greater potential for absorbing and accumulating Cd.

However, plants are prone to toxicity during heavy metal uptake and growth is affected. This greatly limits the efficiency of phytoremediation. With the deterioration of the environment, staple food crops such as rice and maize are also at risk of exceeding the heavy metal content standards. Therefore, enhancing plant tolerance and improving the efficiency of phytoremediation through the addition of different exogenous substances, or converting soil Cd into a non-absorbable form is a valuable approach in agriculture.

### 5.2. Effects of Adding Exogenous Substances to Plants

It has been verified that exogenous supplementation of beneficial elements, plant growth regulators, or nanomaterials can alleviate the Cd stress, thus approaching normal levels of plant height, leaf photosynthesis, and respiration rate [93,230,231,232].

Ca is essential for plant growth and development [233]. When used as an exogenous substance, Ca can reduce Cd-induced physiological and biochemical disorders [234], and also down-regulate Cd accumulation by decreasing the negative charge of the cell membrane surface [235] in *Salix matsudana* [236] and *Fagopyrum esculentum* [237]. As one of the beneficial elements, Se plays an important role in improving plant stress tolerance. Low concentrations of Se can enhance antioxidant capacity and membrane stability and reduce the uptake of heavy metals and the accumulation of ROS [238]. Se has been reported to alleviate Cd stress in rice, oilseed rape, and sunflower by counteracting Cd-induced nutritional changes and reducing oxidative stress [239,240,241]. It is worth noting that Se is like a ‘‘rapier’’ in plants, with low concentrations producing beneficial effects and high concentrations causing stress instead [230,238].

In short, when plants are subjected to Cd stress, if the exogenous elements are metallic elements such as iron (Fe), Ca, and potassium (K), the mitigation mechanism may be because they regulate the biochemical and physiological aspects of the plants, mitigate toxicity, or compete with Cd to the transporter, thereby reducing the amount absorbed by the plant. If non-metallic elements such as boron (B), Se, and Si, are added as exogenous elements, the mitigation mechanism may be because these elements act as nutrients for plants and enhance the tolerance of plants, or they may form complexes with Cd and reduce the uptake of the plants.

Polyamines, along with hormones like 1-naphthaleneacetic acid (NAA) and ABA, serve as plant growth regulators for the modulation of plant development and the augmentation of plant tolerance to Cd [232,242,243]. Wherein, supplementation of exogenous NAA increases the content of Arabidopsis hemicellulose 1, which immobilizes more Cd in the roots [243]. Exogenous application of polyamines, on the other hand, mitigates the adverse effects of Cd contamination on wheat by activating antioxidant enzyme activities [242]. In addition, with the development of technology, nanomaterials and biochar materials have also become ideal candidates for solving Cd pollution. Biogenic hydroxyapatite nanoparticles effectively mitigate the toxicity of Cd to mung beans by adsorbing Cd^2+^ from the environment and forming a protective layer on plant roots [231]. Biochar materials reduce Cd availability and lignocellulosic biochar and herbaceous biochar have a broader range of remediation applications than manure biochar [244].

Of note, in addition to the exogenous substances mentioned above, the addition of melatonin, citric acid, and amino acids can also lead to higher Cd tolerance in plants [245,246,247]. Therefore, exploring more beneficial exogenous substances, forms, and proportions of additions are of practical significance to improve the efficiency of phytoremediation.

## 6. Conclusions and Perspectives

Cd initiates signal transduction cascades in plants. The exposure to Cd-induced stress in Arabidopsis reduced endogenous auxin content. Concurrently, exogenous supplementation with NAA enhanced the fixation of Cd to the cell wall through an elevation in hemicellulose 1 levels in *A. thaliana* [243]. ABA mitigates oxidative stress following exposure to Cd through the ABI5-MYB49-bHLH cascade, activation of the glutathione pathway, and the formation of an apoplastic barrier [232,248]. In contrast to ABA, ethylene amplifies the deleterious effects of Cd on plants in two distinct manners: by augmenting the generation of reactive oxygen species via *RBOHC* or by impeding the establishment of the apoplastic barrier through unidentified pathways [232,248]. Currently, the influences of auxin, ABA, ethylene, and other hormones on Cd tolerance in plants have been initially revealed [232], yet their molecular mechanisms and regulatory networks remain elusive. Given that distinct hormones may elicit contrasting responses under Cd-induced stress, how do they intricately interplay? How do hormone signals crosstalk when encountering ROS or other signals? In addition to the aforementioned circumstances, plant roots may experience heterogeneous Cd stress, characterized by intense pressure on one side and minimal or absent pressure on the other. At this point, plants remodel the root structure and avoid the side with the high level of Cd stress through the RBOH-ROS-growth hormone signaling cascade [249]. The exploration of whether other signaling cascades exist in this process and how they function necessitates further investigation.

A shows the screening of Cd-hyperaccumulator plants and fast-growing woody plants that can accumulate large amounts of Cd. B shows the application of different exogenous substances to plants or genetic modification to make plants have higher Cd tolerance.

**Figure 5:**
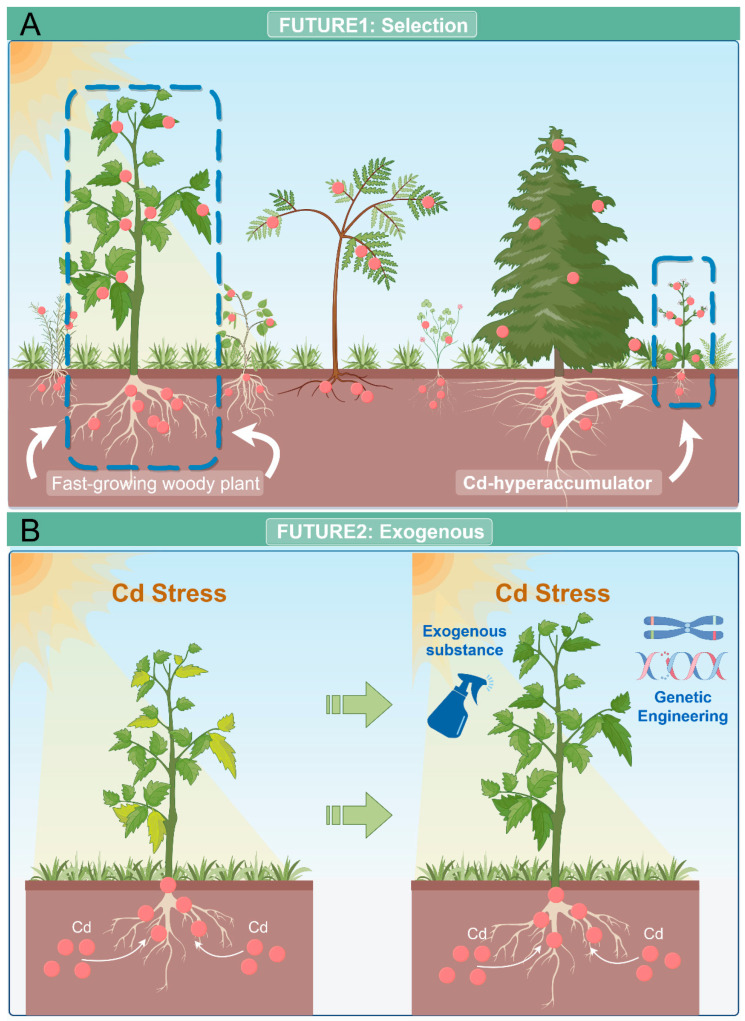
Hot spots for future phytoremediation of Cd pollution. (**A**) Screening of Cd hyperaccumulation-tolerant plants and woody plants that can take up more Cd; (**B**) Enhancement of Cd tolerance in plants by exogenous means (chemical regulators or genetic engineering).

Throughout plant evolution, certain species have developed robust resistance mechanisms as a result of prolonged adaptation to high Cd pollution. Therefore, these highly tolerant plants can be regarded as potential candidates for mitigating excessive Cd pollution in the environment. At present, Cd-hyperaccumulator plants, predominantly wild herbs, are frequently employed in phytoremediation. However, due to their limited aboveground biomass, the overall Cd absorption capacity remains relatively modest. Conversely, fast-growing woody plants, such as poplars and willows, boast larger size, greater biomass, and a swifter growth rate, thereby enabling them to absorb more Cd (Figure 5A). Phytoremediation technology has a broad application prospect in Cd-contaminated soil remediation, but many aspects have not yet been clarified, and for Cd-hyperaccumulator plants, it is worth exploring whether they can improve their growth rate, biomass and accumulated heavy metal content, and stress resistance. For fast-growing woody plants, it is necessary to continue to explore their absorption mechanisms at the molecular level and apply transgenic technology or gene editing to improve the uptake of Cd (Figure 5B).

To optimize the efficiency of Cd pollution phytoremediation, we propose the supplementation of plant regulators (exogenous hormones or polyamines) and beneficial elements to plant nutrition (Figure 5B). Studies have reported that employing this approach confers beneficial effects on plant metabolic pathways and enhances stress resilience [232,236,240,242]. At present, there are fewer studies on the compound addition of multiple substances, and it is worthwhile to explore in depth under what ratio different elements or hormones are added to produce better results. In addition, the application of nanomaterials, biochar materials, and microorganisms can effectively reduce the effectiveness of Cd in the soil, so the combination of nanomaterials/biochar, clumping rhizobial fungi, plant growth-promoting bacteria with Ca, K, B, Si may be an effective way to reduce the uptake of Cd by plants [250]. In conclusion, the discovery of environmentally friendly, cost-effective exogenous substances holds significance for crop and vegetable production.

## Data Availability

The datasets generated during and/or analyzed during the current study are available from the corresponding author on reasonable request.

## Supplementary Materials

The following supporting information can be downloaded at: https://www.mdpi.com/article/10.3390/cells13110907/s1, Table S1: Gene family related to Cd Chelation, accumulation, and detoxification. Table S2: Transcription factors (TFs) families related to Cd uptake, transport, and tolerance. Refs. [251,252,253,254,255,256,257,258,259,260,261,262,263,264,265,266,267,268,269,270,271,272,273,274,275,276,277,278,279,280,281,282,283,284,285,286,287,288,289,290,291,292,293,294,295,296,297,298,299,300,301,302,303,304,305,306,307,308,309,310,311,312,313,314,315,316,317,318,319,320,321,322,323,324,325,326,327,328,329,330,331,332,333,334,335] are cited within.
