# Supplementary material for: The Uptake, Transfer, and Detoxification of Cadmium in Plants and Its Exogenous Effects
Source: Cells. 2024 May 24;13(11):907. doi: 10.3390/cells13110907 (PMC11172145; doi:10.3390/cells13110907)
Supplement: Supplementary file 1 [file cells-13-00907-s001.zip › Table S1.pdf]

Table S1. Gene family related to Cd Chelation, accumulation, and detoxification.

**Gene Family**	**Plant**	**Gene**	**Expression Site**	**Function**	**Reference**
The natural resistance-associated macrophage protein family (NRAMP)	*Spirodela polyrhiza*	*SpNRAMP2*	Roots and fronds	Cd accumulation	[127,128]
		SpNRAMP3	Roots and joint	Cd tolerance	[127,128]
The heavy metal ATPases (The P_{1B}-type ATPases family)	*Oryza sativa* L.	*OsHMA2*	Roots	Cd translocation	[81,251]
		OsHMA3	Roots	Cd sequestration within vacuoles	[45,252]
	Arabidopsis thaliana	*AtHMA1*	-	Cd detoxification	[85]
		AtHMA3	Roots and leaves	Cd tolerance and sequestration within vacuoles	[90,253]
		AtHMA4	Roots	Cd translocation	[88,89]
	Sedum plumbizincicola	*SpHMA1*	Leaves, stems, and roots	Chloroplast Cd exporter	[254]
		SpHMA2	Leaves and roots	Cd translocation and accumulation	[149]
		SpHMA3	Stems and leaves	Cd tolerance and sequestration within vacuoles	[255]
	Noccaea caerulescens (*Thlaspi caerulescens*)	*TcHMA3*	Leaves and roots	Cd tolerance and sequestration within vacuoles	[256]
Gene Family	Plant	Gene	Expression Site	Function	Reference
The heavy metal ATPases /The P_{1B}-type ATPases family (HMAs)	*Populus tomentosa Carr.*	*PtoHMA5*	-	Cd translocation	[257]
	Brassica juncea	*BjHMA4R*	Roots, stems, and leaves	Cd chelation and tolerance	[258]
	Miscanthus sacchariflorus	*MsYSL1*	Whole plant body	Cd tolerance and detoxification	[259]
The ATP-binding cassette transporter family (ABC)	*Oryza sativa* L.	*OsABCC9*	Roots	Cd sequestration within vacuoles	[260]
		OsABCG48	Roots	Cd tolerance	[261]
	Arabidopsis thaliana	*AtABCC1*	Roots and shoots	Cd-PC sequestration within vacuoles	[262]
		AtABCC2	Roots and shoots	Cd-PC sequestration within vacuoles	[262]
		AtABCC3	-	Cd-PC sequestration within vacuoles	[263]
		AtABCC6 (AtMRP6)	Roots	Cd tolerance	[264]
		AtMRP7	Roots and leaves	Cd sequestration within vacuoles	[265]
	Triticum aestivum	*TaABCC13*	-	Cd detoxification	[266]

	Fragaria vesca	*FvABCC11*	Stems and leaves	Cd tolerance	[267]
Gene Family	Plant	Gene	Expression Site	Function	Reference
The placenta-specific 8-domain - containing family (PLAC8)	*Oryza sativa* L.	*OsFWL6*	-	Cd tolerance	[168]
		OsFWL7	Roots	Cd accumulation and tolerance	[168,268]
	Arabidopsis thaliana	*AtPCR1*	-	Cd tolerance	[269]
		AtPCR2	-	Cd tolerance	[269]
		AtPCR9	-	Cd tolerance	[269]
		AtPCR10	-	Cd tolerance	[269]
The metal tolerance protein family (MTPs)	*Taxus media*	*TmMTP1*	-	Cd sequestration within vacuoles	[270]
		TmMTP11	-	Cd sequestration within vacuoles	[270]
	Sedum plumbizincicola	*SpMTP5*	Roots	Cd transport into Golgi	[114]
	Solanum tuberosum L.	*StMTP8*	-	Cd tolerance	[271]
		StMTP9	-	Cd tolerance	[271]
The defensin-like protein family (DEFL)	*Oryza sativa* L.	*OsCAL2*	Roots	Cd chelation	[272]
		OsDEF8	Roots and shoots	Cd unloading and accumulation	[273,274]
		OsThi9	Roots, stems, and seeds	Cd chelation onto cell wall	[275]
	Arabidopsis thaliana	*AtPDF2.6*	Roots	Cd chelation	[276]
	Brassica napus	*BnPDFL*	-	Cd chelation	[277]
Gene Family	Plant	Gene	Expression Site	Function	Reference
The phytochelatin synthase family (PCS)	*Oryza sativa* L.	*OsPCS1*	Roots and shoots	Cd tolerance	[278]
		OsPCS2	Roots	Phytochelatin synthase and Cd accumulation	[279]
		OsPCS5	-	Cd tolerance	[280]
		OsPCS15	-	Cd tolerance	[280]
	Arabidopsis thaliana	*AtPCS1*	-	Cd tolerance	[281,282]
	Triticum aestivum	*TaPCS1*	-	Cd accumulation in shoots	[283]
The cation/calcium superfamily (CaCA)	*Arabidopsis thaliana*	*AtCAX2*	Roots	Cd tolerance sequestration within vacuoles	[36,37]
		AtCAX4	Roots	Cd tolerance	[36,37]
	Arabidopsis halleri	*AhCAX1*	-	Cd tolerance	[284]
	Sedum alfredii	*SaCAX2n*	-	Cd accumulation	[285]
		SaCAX2h	-	Cd accumulation	[285]
	Solanum tuberosum	*STCAX1*	Roots	Cd tolerance	[286]
		STCAX4	Leaves	Cd tolerance	[286]
The cysteine-rich peptide family (CYSTM)	*Digitaria ciliaris*	*DcCDT1*	-	Prevent entry of Cd	[186]
	Iris. Lacteal var. *chinensis*	*IlCDT1*	-	Cd tolerance and accumulation	[287]
Gene Family	Plant	Gene	Expression Site	Function	Reference
The terpene cyclase/mutase family	*Oryza sativa* L.	*OsLCD*	Roots and leaves	Cd accumulation	[95,288]
	Oryza sativa L.	*OsMT-I-Id*	Roots	Cd chelation	[289]

The metallothionein superfamily	*Suaeda salsa*	*SsMT2*	Seeds	Cd tolerance and ROS scavenging	[290]
	Sedum plumbizincicola	*SpMT2*	Roots and Shoots	Cd chelation	[291]
The amino acid/polyamine transporter 2 family	*Oryza sativa* L.	*OsAUX1*	Roots	Cd tolerance	[292]
-	*Oryza sativa* L.	*OsCADT1*	Roots and shoots	Cd tolerance	[293]
-	*Oryza sativa* L.	*OsrgMT*	Leaves and roots	Cd chelation	[294]
-	*Oryza sativa L.*	*OsCLT1*	Roots and stems	GSH homeostasis and Cd tolerance	[295]
-	*Populus euphratica*	*PeANN1*	-	Cd accumulation	[296]
The Nicotianamine synthase Family (NAS)	*Sedum alfredii* Hance	*SaNAS1*	-	Cd tolerance	[297]
The glycosyl hydrolase 16 family	*Populus euphratica*	*PeXTH*	-	Prevent entry and accumulation of Cd	[298]
Gene Family	Plant	Gene	Expression Site	Function	Reference
The protein kinase superfamily	*Arabidopsis thaliana*	*WAKL11*	-	Cd tolerance	[213]
-	*Triticum aestivum* L.	*TaCOPT3D*	Roots	Cd tolerance	[299]
-	*Arabidopsis thaliana*	*AtFC1*	Stems, flowers, leaves, and roots	Cd tolerance	[300]

"-" means unspecified. ROS, reactive oxygen species; PC, phytochelatin; GSH, glutathione.
