# Supplementary material for: The Uptake, Transfer, and Detoxification of Cadmium in Plants and Its Exogenous Effects
Source: Cells. 2024 May 24;13(11):907. doi: 10.3390/cells13110907 (PMC11172145; doi:10.3390/cells13110907)
Supplement: Supplementary file 1 [file cells-13-00907-s001.zip › Table S2.pdf]

Table S2. Transcription factors (TFs) families related to Cd uptake, transport, and tolerance.

Gene Family	Plant species	Gene	Function	Reference
		AtWRKY12	Cd tolerance; Target *AtGSH1*	[194]
		AtWRKY13	Cd tolerance; Target *AtPDR8*	[195,196]
		AtWRKY18	Cd tolerance; Higher rate of H_2S	[197]
	Arabidopsis thaliana	*AtWRKY33*	Cd tolerance; Target *AtATL31*	[133]
		AtWRKY40	Cd tolerance; Higher rate of H_2S	[197]
		AtWRKY45	Cd tolerance; Target *PCS1* and *PCS2*	[191]
WRKY		*AtWRKY60*	Cd tolerance; Higher rate of H_2S	[197]
		PyWRKY48	Cd uptake, accumulation, and tolerance	[301]
	Populus yunnanensis	*PyWRKY71*	Cd tolerance	[302]
		PyWRKY75	Cd tolerance and accumulation	[303]
	Tamarix hispida	*ThWRKY7*	Cd tolerance; Target *ThVHAc1*	[304]
	Sedum alfredii Hance	*SaWRKY7*	Cd stress	[305]
		TaWRKY22	Cd tolerance; Target *TaCOPT3D*	[299]
	Triticum aestivum L.	*TaWRKY70*	Cd tolerance; Target *TaCAT5*	[306]
		TaWRKY74	Cd tolerance	[307]
Gene Family	Plant	Gene	Function	Reference
		GmWRKY142	Cd uptake and tolerance; Target *ATCDT1*, *GmCDT1-1*, and *GmCDT1-2*	[308]
	Glycine max (L.) Merr.	*GmWRKY172*	Cd tolerance and accumulation	[309]
WRKY	*Solanum tuberosum*	*StWRKY6*	Cd tolerance; Target *StCAX1/4*	[286,310]
	Capsicum annuum	*CaWRKY41*	Cd uptake; Enhanced Zn transporter activity	[311]
	Vitis vinifera L.	*VvWRKY2*	Alleviate Cd toxicity	[312]
	Zea mays L.	*ZmWRKY4*	Cd tolerance; Synthesis of antioxidant enzymes.	[313]
		ZmWRKY64	Cd tolerance	[314]
MYB	*Oryza sativa* L.	*OsMYB45*	Cd tolerance	[315]
Gene Family	Plant	Gene	Function	Reference
		AtMYB4	Cd tolerance; Enhance expression of *AtPCS1* and *AtMT1C*	[316]
MYB	*Arabidopsis thaliana*	*AtMYB43*	Cd tolerance; Inhibition of *HMA2*, *HMA3* and *HMA4* expression	[317]
		AtMYB49	Cd accumulation	[192]
		AtMYB75	Cd tolerance; ROS scavenge; Enhance	[318]

			expression of *AtACBP2* and *AtABCC2*	
	Boehmeria nivea L.	*BnMYB2*	Cd tolerance and accumulation	[319]
	Broussonetia papyrifera	*BpMYB1*	Cd uptake and tolerance	[320]
	Tamarix hispida	*ThDIV2 (DIVARICATA)*	Cd tolerance; Target *ThAO1* and *ThAO2*	[321]
	Salicornia brachiata	*SbMYB15*	Cd tolerance	[322]
bHLH	*Arabidopsis thaliana*	*AtbHLH38*	Cd chelation and tolerance; Enhance expression of *AtNAS1* and *At NAS1*	[323]
Gene Family	Plant	Gene	Function	Reference
bHLH	*Arabidopsis thaliana*	*AtbHLH39*	Cd chelation and tolerance; Enhance expression of *AtNAS1* and *At NAS1*	[323]
		AtbHLH104	Cd tolerance	[324]
	Triticum aestivum	*TabHLH094*	Cd tolerance; Form complex with *TaMYC8*	[325]
		TaMYC8	Cd tolerance; Promote *TaERF6* transcription; Regulate ethylene synthesis	[326]
	Glycine max (L.) Merr.	*GmORG3*	Cd tolerance	[327]
	Sorghum bicolor L.	*SbbHLH041*	Cd tolerance; Upregulates *SbEXPA11* expression	[190]
NAC	*Oryza sativa* L.	*OsNAC15*	Cd tolerance	[328]
		OsNAC300	Cd tolerance	[329]
	Arabidopsis thaliana	*ANAC004*	Cd tolerance; Down-regulated expression of *HMA2* and *HMA4*	[193]
		AtNAC102	Cd tolerance	[213]
Gene Family	Plant	Gene	Function	Reference
NAC	*Triticum aestivum*	*TaNAC22*	Cd tolerance	[330]
	Aegilops markgrafii	*AemNAC2*	Cd tolerance	[331]
Heat shock transcription factor family (Hsf)	*Oryza sativa* L.	*OsHsfA4a*	Cd tolerance	[332]
	Solanum lycopersicum L.	*SlHsfA1a*	Cd tolerance; Accumulation of melatonin	[333]
	Triticum aestivum	*TaHsfA4a*	Cd tolerance	[332]
	Sedum alfredii Hance	*SaHsfA4c*	Cd tolerance; Up-regulate the activities of ROS-scavenging enzyme	[334]
-	*Phaseolus vulgaris*	*PvERF15*	Cd tolerance	[335]

"-" means unspecified. H_2S, Hydrogen sulfide.
